# Full Genomic Characterization of a Lentogenic Newcastle Disease Virus Isolated from Farm-Reared Ostriches (*Struthio camelus*) in Northwest China

**DOI:** 10.1128/genomeA.01590-16

**Published:** 2017-02-09

**Authors:** Shanhui Ren, Chongyang Wang, Xiaolong Gao, Xue Zhang, Xiangwei Wang, Yanqing Jia, Yanhong Wang, Xinglong Wang, Zengqi Yang

**Affiliations:** Department of Avian Disease, College of Veterinary Medicine, Northwest A & F University, Yangling, Shaanxi, People's Republic of China

## Abstract

To our knowledge, our study is the first to report the whole-genome sequence of an ostrich-origin Newcastle disease virus (NDV) isolate, abbreviated as Ostrich/SX-01/06. Phylogenetic analysis revealed that this isolate belongs to the subgenotype c in class II. The identification of the complete genome will provide useful information regarding ostrich diseases, especially NDV.

## GENOME ANNOUNCEMENT

Newcastle disease (ND) is one of the most highly pathogenic viral diseases of avian species (1). ND virus (NDV), the causative pathogen, is also known as avian paramyxovirus type 1 (APMV-1) and belongs to the genus *Avulavirus*, family *Paramyxoviridae*, order *Mononegavirales* (2).

NDV has a wide host range, and it has been demonstrated in at least 250 species in most orders of birds (1). The African ostrich is the largest bird that also serves as a susceptible host of NDV (3). However, the roles of these birds in the ecological evolution of NDV are largely unknown. Furthermore, the complete genomic sequence of ostrich-origin NDV has yet to be published on GenBank.

Two tissue samples from different organs, including liver, lung, heart, spleen, and kidney, were collected from ostriches less than 4 months old at the end of October 2006. After three series of passages in 10-day-old specific-pathogen-free (SPF) chicken embryos, an NDV was isolated from an ostrich and designated NDV/Ostrich/China/Shaanxi-01/2006, abbreviated Ostrich/SX-01/06. Allantoic fluids were collected and stored in the Laboratory of Avian Diseases for use in subsequent analyses. In this study, isolate Ostrich/SX-01/06 was subjected to plaque purification three times by using an agar overlay on chicken embryo fibroblast cells. Total RNA was extracted from allantoic fluids by using RNAisoPlus reagent (TakaRa Biotechnology, Dalian, China). cDNA was synthesized using the PrimeScript first-strand cDNA synthesis kit (TakaRa Biotechnology) with random hexamers, according to the manufacturer’s instructions. Ten overlapping fragments covering the full-length genome of the NDV isolate were amplified by reverse transcription-PCR (RT-PCR). The nucleotide and amino acid sequences of the isolate were assembled and aligned using the DNAStar software suite (version 3.1; DNAStar, Madison, WI, USA). This ostrich-origin NDV genome, with 15,192 nucleotides (nt) in length, comprised six open reading frames (ORFs) in the order 3′-NP-P-M-F-HN-L-5′ and followed the “rule of six,” which is a common rule relied on by other *Paramyxoviridae* members (4).

The pathogenicity index of intracerebral pathogenicity index (ICPI) is 0.1, indicating that Ostrich/SX-01/06 is a letogenic NDV strain. However, the isolate contained a motif of -^112^R-R-Q-R-R-F^117^- at the F_o_ cleavage site, which is a typical molecular characterization of a virulent (velogenic or mesogenic) strain. Coincidentally, several reported NDVs, especially the pigeon paramyxovirus type 1 (PPMV-1) isolates with typical virulent cleavage site motifs were not always confirmed by classical OIE assessment criteria for pathogenicity (5–8). Apparently, these findings suggested that the F cleavage site is required but is not the sole determinant of viral virulence for some NDV isolates to become virulent. Further studies should investigate whether the virulence of Ostrich/SX-01/06 can also be determined by other factors, which can be examined by using a platform of reverse genetic systems.

To determine the genetic relationship, a phylogenetic tree was constructed on the basis of the complete sequence (1,662 bp) (9, 10) using the MEGA 6.02 software with the Kimura two-parameter model and the maximum likelihood (ML) method (11). The results showed that Ostrich/SX-01/06 isolate was clustered in subgenotype c in genotype.

### Accession number(s).

The complete genome sequence of Ostrich/SX-01/06 has been deposited in GenBank under accession number KU373026.

## References

[1] Alexander DJ 2001 Gordon memorial lecture. Newcastle disease. Br Poult Sci 42:5–22. doi:10.1080/713655022. 11337967

[2] Afonso CL, Amarasinghe GK, Bányai K, Bào Y, Basler CF, Bavari S, Bejerman N, Blasdell KR, Briand FX, Briese T, Bukreyev A, Calisher CH, Chandran K, Chéng J, Clawson AN, Collins PL, Dietzgen RG, Dolnik O, Domier LL, Dürrwald R, Dye JM, Easton AJ, Ebihara H, Farkas SL, Freitas-Astúa J, Formenty P, Fouchier RA, Fù Y, Ghedin E, Goodin MM, Hewson R, Horie M, Hyndman TH, Jiāng D, Kitajima EW, Kobinger GP, Kondo H, Kurath G, Lamb RA, Lenardon S, Leroy EM, Li CX, Lin XD, Liu L, Longdon B, Marton S, Maisner A, Muhlberger E, Netesov SV, Nowotny N, et al. 2016 Taxonomy of the order *Mononegavirales*: update 2016. Arch Virol 161:2351–2360. doi:10.1007/s00705-016-2880-1. 27216929 PMC4947412

[3] Alexander DJ 2000 Newcastle disease in ostriches (*Struthio camelus*)—a review. Avian Pathol 29:95–100. doi:10.1080/03079450094117. 19184794

[4] Kolakofsky D, Roux L, Garcin D, Ruigrok RW 2005 Paramyxovirus mRNA editing, the “rule of six” and error catastrophe: a hypothesis. J Gen Virol 86:1869–1877. doi:10.1099/vir.0.80986-0. 15958664

[5] Dortmans JC, Rottier PJ, Koch G, Peeters BP 2010 The viral replication complex is associated with the virulence of Newcastle disease virus. J Virol 84:10113–10120. doi:10.1128/JVI.00097-10. 20660202 PMC2937822

[6] Dortmans JC, Koch G, Rottier PJ, Peeters BP 2009 Virulence of pigeon paramyxovirus type 1 does not always correlate with the cleavability of its fusion protein. J Gen Virol 90:2746–2750. doi:10.1099/vir.0.014118-0. 19641043

[7] Rui Z, Juan P, Jingliang S, Jixun Z, Xiaoting W, Shouping Z, Xiaojiao L, Guozhong Z 2010 Phylogenetic characterization of Newcastle disease virus isolated in the mainland of China during 2001–2009. Vet Microbiol 141:246–257. doi:10.1016/j.vetmic.2009.09.020. 19833457

[8] Liu H, Zhang P, Wu P, Chen S, Mu G, Duan X, Hao H, Du E, Wang X, Yang Z 2013 Phylogenetic characterization and virulence of two Newcastle disease viruses isolated from wild birds in China. Infect Genet Evol 20:215–224. doi:10.1016/j.meegid.2013.08.021. 23999544

[9] Diel DG, da Silva LH, Liu H, Wang Z, Miller PJ, Afonso CL 2012 Genetic diversity of avian paramyxovirus type 1: proposal for a unified nomenclature and classification system of Newcastle disease virus genotypes. Infect Genet Evol 12:1770–1779. doi:10.1016/j.meegid.2012.07.012. 22892200

[10] Dimitrov KM, Ramey AM, Qiu X, Bahl J, Afonso CL 2016 Temporal, geographic, and host distribution of avian paramyxovirus 1 (Newcastle disease virus). Infect Genet Evol 39:22–34. 26792710 10.1016/j.meegid.2016.01.008

[11] Tamura K, Peterson D, Peterson N, Stecher G, Nei M, Kumar S 2011 MEGA5: molecular evolutionary genetics analysis using maximum likelihood, evolutionary distance, and maximum parsimony methods. Mol Biol Evol 28:2731–2739. doi:10.1093/molbev/msr121. 21546353 PMC3203626

